# Ecological and Health Risks Assessment of Potentially Toxic Metals and Metalloids Contaminants: A Case Study of Agricultural Soils in Qatar

**DOI:** 10.3390/toxics9020035

**Published:** 2021-02-12

**Authors:** Mohammed Alsafran, Kamal Usman, Hareb Al Jabri, Muhammad Rizwan

**Affiliations:** 1 Department of Biological and Environmental Sciences, College of Arts and Sciences, Qatar University, Doha 2713, Qatar; m.alsafran@qu.edu.qa; 2 Agricultural Research Station, Qatar University, Doha 2713, Qatar; h.aljabri@qu.edu.qa; 3 Office of Academic Research, Qatar University, Doha 2713, Qatar; 4 Microelement Research Center, College of Resources and Environment, Huazhong Agricultural University, Wuhan 430070, China; m.rizwan110@hotmail.com

**Keywords:** toxic metals, arsenic (As), carcinogenic risk, agricultural soil, health and ecological risk assessment

## Abstract

In recent years, Qatar has witnessed exponential growth in the human population, urbanization, and increased anthropogenic activities, including agriculture. Potentially toxic environmental contaminants, including metals and metalloids, are commonly found in emerging economies. At high concentrations, elements such as As, Cr, and Ni can be hazardous and may lead to various health problems in humans, including cancer. The current study measured As, Cd, Cr, Cu, Ni, Pb, V, and Zn concentrations in agricultural soils. Pollution levels and potential negative impacts on human and environmental health were determined using the United States Environmental Protection Agency (USEPA) standard methodologies. According to the study’s findings, the studied element concentrations descended in the following order: Zn > Cr > V > Ni > As > Cu > Pb > Cd. Of these, As (27.6 mg/kg), Cr (85.7 mg/kg), Ni (61.9 mg/kg), and Zn (92.3 mg/kg) concentrations were higher than average world background levels. Each of these elements also had an enrichment factor (EF > 1), indicating their anthropogenic origin. The combined pollution load index (PLI > 1) and geo-accumulation index (Igeo) range values of −0.2–2.5 further indicated that the soil was up to 58% polluted. However, the ecological risk factor (Er ≤ 40.6) and potential ecological risk index (PERI = 79.6) suggested low ecological risk. A human health risk evaluation showed that only As, with a hazard index (HI) of 1.3, posed a noncarcinogenic risk to infants. Additionally, As, Cr, and Ni, with total carcinogenic risk (TCR) values of 1.18 × 10^−4^ and 2.06 × 10^−4^ for adults and children, respectively, proved carcinogenic to both age groups. The elements’ carcinogenic risk (CR) potential descended in the following order: Ni > As > Cr. Additionally, for both adults and children, oral ingestion is the most likely exposure pathway. Our findings support the need for closer monitoring of potentially toxic metals and metalloids levels in cultivated soils and farm produce in Qatar. Reducing the elements’ bioavailability in soil and developing innovative remediation technologies is needed to limit potential risks to human health. Further studies on As, Cr, and Ni gastrointestinal bioaccessibilities are needed to fully understand the effects after long-term exposure and the cancer-causing potential of these elements over a lifetime.

## 1. Introduction

In June 2017, Saudi Arabia, U.A.E., Bahrain, and Egypt, imposed a land, sea, and air blockade on Qatar. Since the economic sanction, there has been a massive increase in agricultural activities to boost local food production, cushion the blockade’s effect, and ensure future food security and safety. The state has also introduced new enabling policies through public–private partnerships, infrastructural incentives, and increased research funding. There are currently close to 2000 farms in Qatar, with over 200 being in the open, irrigated areas that grow different kinds of vegetables during the winter [1]. Given the soil’s nature, farmers adopt different agricultural practices to enhance its physical, biological, and chemical properties. Amending soil using mature compost, ashes, biochars, and imported soils are common. Using soil amendments improves soil fertility, increases yield, and often offers protection against pathogens by enhancing soil nutrients and important microbes [2,3]. Over the years, there has been a remarkable increase in local food production, especially vegetables, poultry, and dairy products. In 2018, the Ministry of Municipality and Environment announced that Qatar achieved 82–98% self-sufficiency in poultry products and dates and was expected to attain 90–100% self-sufficiency in fresh vegetables and other food products by the end of the year 2020 [4].

However, the boost in agriculture and other activities, including construction, oil and gas, and waste generation, due to exponential population growth, potentially increases environmental pollution, posing significant concerns for human and ecological health [4,5]. Potentially toxic elements such as arsenic (As), copper (Cu), chromium (Cr), and lead (Pb) are dangerous to the environment and humans [6,7]. These may find their way into the human body by ingesting contaminated food products or direct inhalation and skin exposure [8]. Humans’ long-term exposure to high levels of such pollutants can result in various health problems, including cancer [5,9].

Previously, the occurrence of potentially toxic elements, including As, cadmium (Cd), Cu, Cr, nickel (Ni), and Pb, in noncultivated soil in Qatar, has been reported [10,11]. Peng et al. (2016) [12] found extreme concentrations of Cr, Cu, and Ni in agricultural soil. The same study also reported varying levels of other potentially toxic metals, including As, Pb, and zinc (Zn). However, based on standard guidelines, there are no studies about how the reported metals are associated with health risks, particularly in humans. Since the renewed local agricultural production efforts in Qatar, no study about the impact of metals in cultivated soil and food products on human health has been conducted to the best of our knowledge.

Various parameters to assess metals and metalloids’ human health and ecological risks in soil and sediment are used. The biological, chemical and physical properties of soils and the elements exposure routes vary widely. Therefore, robust parameters are used to evaluate toxicant pollution levels and associated public health risks [13,14]. In recent times, contamination factors (CF), geo-accumulation indexes (Igeo), and enrichment factors (EF) have become popular for detecting pollution and indicating potential pollution sources [15]. At the same time, cancer risk (CR) and hazard index (HI) are measurements used to determine carcinogenic and noncarcinogenic risks to humans, respectively. CR estimates the probability of adults or children developing cancer after exposure to potentially carcinogenic elements. HI summarizes all the noncarcinogenic hazards of potentially toxic elements. Several studies have linked the presence of these elements in soil to increased agricultural activities such as the use of pesticides, animal manure, and other soil amendments and their associated human health risks [4].

The present study aims to characterize agricultural soil in selected cultivated areas in northern and central Qatar; evaluate the degree of As, Cd, Cr, Cu, Ni, Pb, V, and Zn pollution; assess potential ecological and human health risks (adults and children). As noted above, due to Qatar’s small land size and Qatar soil’s arable nature, hydroponic-based cultivation methods are the most widely practiced. Therefore, sampling locations with a concentration of open, irrigated farms were chosen. Our findings, coming when the country is prioritizing national food security and safety, are significant. They will help shape local agricultural production policies and inform the creation of legislation and pollution management strategies to protect the environment and human health. They will also support sustainable development based on the diversity of global climate, regional/country-specific determinants of policy formulation, and political will towards environmental sustainability for health.

## 2. Materials and Methods

### 2.1. The Study Areas, Sampling, and Processing

**Figure 1:**
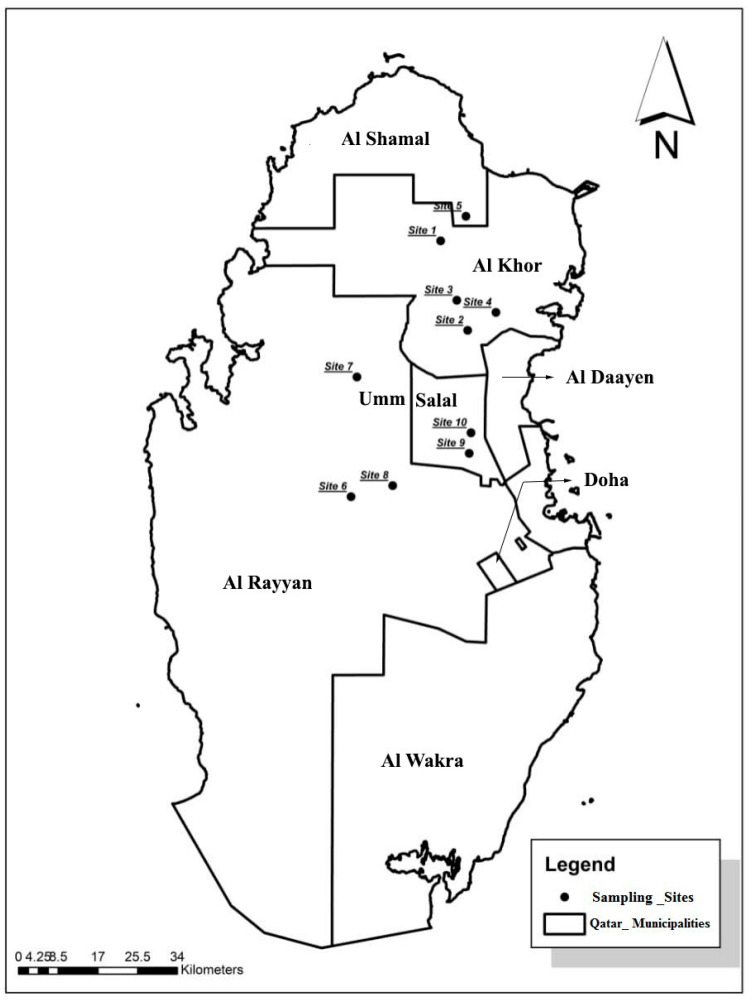
Sampling locations.

Qatar is a low-lying peninsula characterized by the arch’s surface expression, one of the Arabian plate’s most notable structural characteristics. Loose sand and pebbles broken off the outcropping limestone cover it. While little is known about Qatar’s lithology, most of the country’s surface lies on Cenozoic strata primarily composed of limestone and clay, both of which are not fully exploited. Previous studies on the remediation of potentially toxic metals and metalloids, including As, Cd, and Cr, found that the concentrations of these elements in Qatar’s natural soil were often below the detection limit. Therefore, to the best of our knowledge, there is no geological evidence that suggests that lithological events increase metal concentration, particularly As, Cr, and Ni. The sites chosen for this study are open irrigated farms spread across the northwestern and central parts of Qatar (Figure 1). From January to April 2020, 50 soil samples from 10 separate locations, all within 70 km of Doha city center, were gathered. 

Sampling locations were chosen randomly, and their coordinates were recorded (see Table 1). From the chosen sites, cultivated produce included spinach, parsley, lettuce, silk, dill, coriander, onion, Rocca, mint, and vegetable silk. Common soil amendments used to improve soil fertility were compost manure, peat moss, and other imported soils. A clean auger was used at each location to perform sampling about 30 cm below the soil’s surface approximately every 40 m. A total of 50 soil samples were transported to the laboratory, air, and oven-dried (at 60 °C temperature). Before soil sieving, tweezers were used to remove visible plant material. The dried samples were sieved to remove fractions > 2.0mm. Fractions ˂ 2.0mm were further crushed into finer particles using an agate mortar and sieved through a 0.25 mm mesh to obtain elemental analysis samples.

### 2.2. Determination of the Soil Physical and Chemical Properties 

We determined soil physicochemical parameters, including pH, electrical conductivity (EC), total carbon (TC), total nitrogen (TN), and soil ionic contents. We measured pH using a portable digital pH meter (Mettler Toledo, FE20 ATC, Schwerzenbach, Switzerland) and EC (dS m-^1^) using an inductive electromagnetic device (Mettler Toledo, S230 SevenCompact, Schwerzenbach, Switzerland). TC and TN were analyzed using a CHNS/O analyzer (Perkin Elmer, Series II 2400 CHNS/O Elemental Analyzer, Boston, MA, USA). All analyses were performed per equipment or instrument manufacturer’s guides and procedures, and as previously reported [10,11]. Soil ionic contents (cations and anions) were extracted in water and analyzed using Ion Chromatography (Metrohm, MagIC Net 3.3, Herisau, Switzerland). Briefly, 10 g of each sample was mixed with 50 mL water and shaken for 24 h. The extracted samples were transferred into new tubes and injected into the IC instrument to determine analyte concentrations. All sample analyses were performed in replicates, and averages of the acquired data were reported.

### 2.3. Metals Quantification

Sieved soil samples were prepared for microwave-assisted digestion of metals using analytical grades of hydrochloric acid (HCl), nitric acid (HNO_3_), and hydrogen fluoride (HF). Before digestion, all tubes and other glassware were soaked, thoroughly washed with HCl, and repeatedly rinsed with deionized water. A calibrated analytical balance was used to weigh 0.25 g of each soil sample. Afterward, 6 mL HCl, 2 mL HNO_3_, and 2 mL HF were added to a total volume of 10 mL of each sample, gently swirled, and transferred into the microwave digester, Buck Scientific (Master Series HP-40), at alternating temperature cycles. After the completed digestion cycles, solutions were moved to 150 mL flasks to be cooled and filtered. Each sample’s solutions were then topped off with deionized water to create a final volume of 150 mL. 

After digestion, the concentrations of As, Cd, Cu, Cr, Pb, Ni, V, and Zn were determined by directly injecting the samples into an Inductively Coupled Plasma Mass Spectrometer (ICP-MS) (Nex Ion 300D). According to the USEPA classification and previous reports on such metals in Qatari soil [12,16], all analytes were chosen based on public health risks [10,11]. For result validation, quality assurance and control procedures such as Reagent blanks, lab duplicates at every 10th sample, and the National Institute Standard for soil (2709a) were used during analyses. The % recovery of all analyzed metals in certified material were between 91% and 109%. Additionally, for all elements, concentrations (mg/kg) were below detection limit in the reagents blanks, and corresponds to the actual samples concentrations with slight variations (within ±1.4 mg/kg) in the lab duplicates.

### 2.4. Contamination Factor (CF)

The CF of all the analyzed metals were determined using soil quality standard guidelines for environmental and human health protection and the equation below [17,18],

$${\mathrm{CF}}_{n}=\frac{C_{\mathrm{sample}}}{C_{\mathrm{background}}}$$

where ${\mathrm{CF}}_{n}\;$is the contamination factor of each metal, $C_{\mathrm{sample}}$ is determined metal concentrations, and $C_{\mathrm{background}}$ is metal background concentration (Supplementary Table S6).

### 2.5. The Pollution Load Index (PLI)

The PLI indicates combined or mutual pollution effects of all the metals found at different sampling sites [19]. The PLI was computed as mean CF values of metals n using the below equation,

$$\mathrm{PLI}=\left({\mathrm{CF}}_{1}\times {\mathrm{CF}}_{2}\times {\mathrm{CF}}_{3}\ldots \times {\mathrm{CF}}_{n}\right)\frac{1}{n}$$

where PLI is the pollution load index, n is the number of analyzed metals, and ${\mathrm{CF}}_{n}$ is the contamination factor of metals. A PLI value of 1 or > 1 indicates pollution, while values ˂ 1 suggest metal levels being near background levels [20].

### 2.6. Geo-Accumulation Index ($I_{geo}$) and Enrichment Factor (EF)

The use of $I_{\mathrm{geo}}$ was introduced by Muller (1969) [19] and has become an important index for assessing pollution levels in soil and sediment samples [9,21]. To calculate $I_{\mathrm{geo}}$, the equation below was used,

$$I_{\mathrm{geo}}=\log_{2}\left(\frac{C_{\mathrm{sample}}}{1.5\;\times {\;C}_{\mathrm{background}}}\right)$$

where $C_{\mathrm{sample}}$ is the determined metal concentration and $C_{\mathrm{background}}$ represents the average geochemical background values of determined metal concentrations. Average geochemical background concentration values are provided in Supplementary Table S6 [22,23]. The $I_{\mathrm{geo}}$ values 3 < $I_{\mathrm{geo}}$ ≤ 4; 2 < $I_{\mathrm{geo}}$ ≤ 3; 1 < $I_{\mathrm{geo}}$ ≤ 2; 0 < $I_{\mathrm{geo}}$ ≤ 1; $I_{\mathrm{geo}}$ < 0 indicate heavy, moderate to heavy, moderate, not polluted to moderate, and not polluted soils, respectively.

The enrichment factor (EF) provides essential information for evaluating the degree and source of contamination [14]. EF values of elements in the soil were computed using the equation below,

$$\mathrm{EF}=\frac{{\left(C_{x}/C_{\mathrm{ref}}\right)}_{\mathrm{sample}}}{{\left(C_{x}/C_{\mathrm{ref}}\right)}_{\mathrm{background}}}$$

where EF is the metal enrichment factor; x, $C_{x}$ is the determined concentration of the metal x in soil (mg kg^−1^), $C_{\mathrm{ref}}$ is the reference metal concentration in soil (mg/kg), and $C_{x}/C_{\mathrm{ref}}$ is the ratio of the determined metal concentration to that of the background concentration of reference metal.

EF values are used to evaluate contamination levels and determine whether possible sources were natural, such as the weathering process, or anthropogenic, such as agriculture and other industrial activities. It involves the normalization of sediments relative to reference elements, for example, scandium (Sc), titanium (Ti), and manganese (Mn) [24], iron (Fe), and aluminum (Al) [25]. It is worth mentioning that the concentrations of other elements from the same samples, including Al, Co, and Mn, were determined but not reported. Therefore, Mn concentration from the same sample was used as the reference element as most symbolized by Loska [26] and Ahamad et al. (2020) [27]. 

### 2.7. Ecological Risk Analysis

This study evaluated the ecological risks related to the analyzed metals using parameters, the possible risk of a given metal (Er), and the potential ecological risk index (PERI) using the below equations as proposed in [28] and widely used in several similar studies [29,30],

$$C_{f}^{i}=C_{D}^{i}/C_{r}^{i}$$

$$\mathrm{Er}=T_{r}^{i}\times C_{f}^{i}$$

$$\mathrm{PERI}=\sum_{i=1}^{m}{\mathrm{Er}}^{i}$$

where $C_{f\;}^{i}$is the contamination factor; $C_{D}^{i}$ is the mean concentration of the metal; $C_{r}^{i}$ is the pre-industrial reference values (PRV) of the sediments; Er is the potential risk of an individual metal; $T_{r}^{i}$ stands for toxic-response factor (TRF) for a given metal; PERI is the sum of the potential risk of an individual metal, and m represents the number of the metals. In this study, the ecological risk index classifications were based on [28]. Hakanson classified the Er as follows: Er < 40, 40 ≤ Er < 80, 80 ≤ Er < 160, 160 ≤ Er < 320, and Er ≥ 320 as low, moderate, considerable, high, and very high potential ecological risks, respectively; while PERI was classified as follows: RI < 150, 150 ≤ RI < 300, 300 ≤ RI < 600, and RI ≥ 600 as low, moderate, considerable, and very high ecological risks, respectively. 

### 2.8. Health Risk Analysis 

For this work, the human health risks related to the analyzed metals were evaluated as described in [9,31]. Additionally, the United States Environmental Protection Agency (USEPA) regulations for assessing both noncarcinogenic and carcinogenic risks in human children and adults were used [32].

The noncarcinogenic risks for humans via dermal contact, inhalation, and ingestion were analyzed using the inputs$\;{\mathrm{ADD}}_{\mathrm{inhalation}}$, ${\mathrm{ADD}}_{\mathrm{dermal}}$, and ${\mathrm{ADD}}_{\mathrm{ingestion}}$ which corresponded to mouth and nose inhalation, skin exposure, and oral intake (mg/kg/day), respectively. The human cancer risk of being exposed to the analyzed metals via the inhalation pathway estimates the cumulative probability of individuals developing cancer over time after exposure to a potential carcinogen (i.e., the metal). The following equations were used:

$${\mathrm{ADD}}_{\mathrm{ingestion}}=\frac{C_{\mathrm{soil}\;\times \;\mathrm{IngR}\;\times \;\mathrm{EF}\;\times \;\mathrm{ED}\;}}{{\mathrm{BW}}_{A\;}\times {\;\mathrm{ET}}_{A}}\;\times \;10^{-6}$$

$${\mathrm{ADD}}_{\mathrm{dermal}}=\frac{C_{\mathrm{soil}\;\times {\;\mathrm{ESA}}_{s}\times \;\mathrm{ABS}\;\times {\;\mathrm{AF}}_{s}\times \;\mathrm{EF}\;X\;\mathrm{ED}\;}}{{\mathrm{BW}}_{A\;}\times {\;\mathrm{ET}}_{A}}\;\times \;10^{-6}$$

$${\mathrm{ADD}}_{\mathrm{inhalation}}=\frac{C_{\mathrm{soil}\;\times \;\mathrm{InhR}\;\times \;\mathrm{EF}\;X\;\mathrm{ED}\;}}{{\mathrm{BW}}_{A\;}\times {\;\mathrm{ET}}_{A}\;\times {\mathrm{EF}}_{p}}$$

$$\mathrm{HI}=\sum^{\;}{\mathrm{HQ}}_{i}=\sum^{\;}\frac{{\mathrm{ADD}}_{i}}{{\mathrm{RfD}}_{i}}$$

$${\mathrm{CR}}_{\mathrm{inhalation}}=\frac{C_{\mathrm{soil}\;\times \;\mathrm{InhR}\;\times \;\mathrm{EF}\;X\;\mathrm{ED}\;}}{{\mathrm{BW}}_{A\;}\times {\;\mathrm{ET}}_{\mathrm{ca}}\;\times {\mathrm{EF}}_{p}}\times {\;\mathrm{SF}}_{\mathrm{inhalation}}$$

$${\mathrm{CR}}_{\mathrm{dermal}}=\frac{C_{\mathrm{soil}\;\times {\;\mathrm{ESA}}_{s}\times \;\mathrm{ABS}\;\times {\;\mathrm{AF}}_{s}\times \;\mathrm{EF}\;X\;\mathrm{ED}\;}}{{\mathrm{BW}}_{A\;}\times {\;\mathrm{ET}}_{A}}\;\times \;10^{-6}\times {\;\mathrm{SF}}_{\mathrm{dermal}}$$

$${\mathrm{CR}}_{\mathrm{ingestion}}=\frac{C_{\mathrm{soil}\;\times \;\mathrm{IngR}\;\times \;\mathrm{EF}\;\times \;\mathrm{ED}\;}}{{\mathrm{BW}}_{A\;}\times {\;\mathrm{ET}}_{\mathrm{ca}}}\;\times \;10^{-6}\times {\;\mathrm{SF}}_{\mathrm{ingestion}}$$

$$\mathrm{TCR}=\sum^{\;}\left({\mathrm{CR}}_{\mathrm{ingestion}}+{\mathrm{CR}}_{\mathrm{dermal}}+{\mathrm{CR}}_{\mathrm{inhalation}}\right)$$

where ADD is the average daily dose and $C_{\mathrm{soil}\;}$(mg/kg), InhR (m^3^/day), and IngR (mg/kg) are metal concentrations in the soil and inhalation and ingestion rates, respectively. Exposure frequency and duration are represented as EF (day/year) and ED (year), respectively. ${\mathrm{ET}}_{A}$, ${\mathrm{BW}}_{A\;},{\;\mathrm{ESA}}_{s\;},\;$and ${\mathrm{AF}}_{s}$ stand for the average time of exposure (day), exposed body weight (kg), exposed skin area (cm^2^), and adherence factor (mg/cm^2^), respectively. ${\mathrm{EF}}_{p}$ represents the particle emission factor (m^3^/kg), whereas ${\mathrm{RfD}}_{i}$ stands for the reference dose (mg/kg/day) and i the number of exposure pathways. HQ represents the hazard quotient, while HI is the calculated reference dose and hazard quotient. All result interpretations and health risk analyses were based on USEPA guidelines and were previously used in similar studies. The definitions and reference values for parameters used to estimate average daily intake (ADI) for noncarcinogenic and carcinogenic risk, metals reference doses (RfD), and the cancer slope factors (SF) for As, Cr, and Ni are provided in the Supplementary Data Tables S1–S3.

### 2.9. Statistical Analysis

Statistical analysis of the dataset was conducted using Microsoft Office Excel and SPSS 16.0 (SPSS Inc., Chicago, IL, USA). Variable distribution was studied using descriptive statistics and Pearson’s correlation coefficients. 

## 3. Results and Discussion

### 3.1. The Soil Properties

The results of the soil physicochemical parameters pH, EC, TN, TC, and ionic contents are provided in Supplementary Tables S4 and S5. Across all sampling locations, the pH and EC (dS m^−1^) values ranged from 7.1 ± 0.0 to 7.7 ± 0.5 and 57 ± 0.0 to 1158 ± 2.68, respectively, and the TC and TN values ranged from 6.3 ± 0.8 to 8.9 ± 1.3 and 0.2 ± 0.0 to 0.4 ± 0.0, respectively (Supplementary Table S4). The ionic content measurements showed noticeably high sodium and chloride ion concentrations (mg/kg) with the maximum being 10,407.2 ± 2736.7 and 16,327.5 ± 4492.9, respectively (Supplementary Table S5). Except for TC, the values of all physical and chemical parameters were within previously reported ranges and translated to the general soil properties in Qatar: neutral pH, sandy-saline, and rich in calcium and magnesium ions [7,12]. The relatively high TC (%) levels, ranging from 4.7 ± 0.1 to 8.9 ± 1.2 can be attributed to the carbonates in the studied soils, especially since no previous decarbonization was performed to estimate organic carbon contents. Other factors are the widespread application of soil amendments, such as composted manure and biochars, in the sampled areas [33]. It is worth noting that these areas have witnessed an unprecedented use of large quantities of soil amendments in the last three years.

### 3.2. Metals Concentrations, Descriptive Statistics, and Correlation Analysis

Descriptive statistics of the metal concentrations across all sampled locations were performed, and the results are provided in Supplementary Table S6. The concentrations of V, Cr, Ni, Zn, Cu, As, Cd, and Pb in soils varied from 46.7 to 120.6, 39.5 to 148.1, 24.1 to 131.2, 35.8 to 168.8, 11.6 to 44.9, 14.3 to 52.3, 0.1 to 0.7, and 5.9 to 34.3 mg/kg, respectively, with mean ± SD concentrations of 75.4 ± 20.1, 85.7 ± 24.4, 61.9 ± 29.1, 92.4 ± 30.6, 25.6 ± 7.2, 27.6 ± 9.7, 0.2 ± 0.1, and 18.2 ± 7.1 mg/kg, respectively. Overall, the metal concentrations descended as follows: Zn > Cr > V > Ni > As > Cu > Pb > Cd. Our result is consistent with the finding of Peng et al. (2016) [12], where high concentrations of As, Cr, Ni, and Zn were reported in Qatari agricultural soils. At such concentrations, the levels of As, Ni, Cr, and Zn in the study area soils are noticeably higher than the geochemical background concentrations, being approximately 4.0, 2.1, 1.4, and 1.3 times higher than their corresponding background values, respectively. According to Kabata-Pendias and Mukherjee [22], the standard background concentrations (mg/kg) for As, Ni, Cr, and Zn are 6.8, 59.5, 29, and 70, respectively. Therefore, our findings suggest that these elements’ elevated levels in the study areas could be due to increased agricultural activities.

The coefficient of variation (CV) can also indicate the source of metal pollution. Mean metal concentrations with a low CV are generally due to natural resources, while elements with a high CV result from anthropogenic activities [34,35]. The CVs for V, Cr, Ni, Zn, Cu, As, Cd, and Pb were 26.7%, 28.5%, 46.9%, 33.1%, 28.1%, 35.2%, 57.9%, and 39.1%, respectively (Supplementary Table S6). The CV of the elements concentrations in the soils descended as follows: Cd > Ni > Pb > Zn > As > Cr > Cu > V. The CV of all the studied metals ranged from 25% to 50%, demonstrating a moderate degree of variation in the metal pollution in the investigated areas. These findings suggest that the soil in the study areas was severely affected by distinct inputs related to anthropogenic activities and natural and external influences [9,35]. 

The relationships between different metals were analyzed, and the correlation coefficients are shown in Supplementary Table S6. The results indicate a mixed relationship between elements. A positive correlation exists for V-Cr-Ni-Cu-Cd-Zn-Pb, indicating that these metals may come from similar sources. However, the correlation of As with all the studied metals was negative, suggesting that the studied areas have a unique origin. Previous research shows that Cr, Ni, and V in soil primarily comes from parent materials [14,36]. 

In contrast, increased levels of other metals are more likely to occur because of anthropogenic sources. For instance, agricultural activities, including phosphate fertilizers and pesticides, contribute to high Pb and Zn levels [36]. Additionally, coal fly ashes and the combustion of petroleum products result in the atmospheric deposition of As increasing metal levels in agricultural soils [37,38].

### 3.3. Metals Contamination Level and Potential Ecological Risks Assessment 

#### 3.3.1. Enrichment Factor (EF)

**Figure 2:**
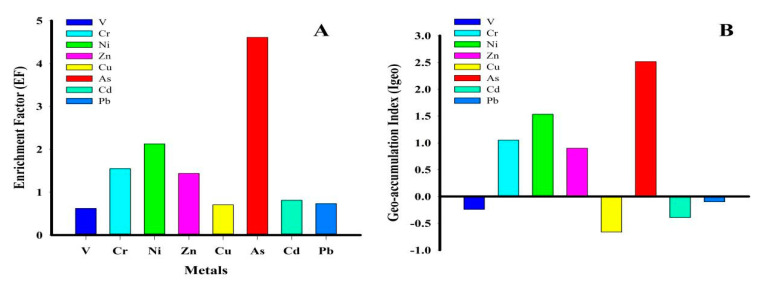
(**A**) Metals enrichment factors (EF); (**B**) geo-accumulation index (Igeo).

The EF can be used to assess the extent of metal pollution in soil due to human activities. In general, an EF < 1.0 specifies that an element is made up of crustal materials or was created by natural weathering processes, whereas an EF > 1.0 implies anthropogenicity [35]. Figure 2A displays EF values of V, Cr, Ni, Zn, Cu, As, Cd, and Pb that range from 0.5 to 0.9, 0.9 to 2.9, 1.5 to 2.7, 0.8 to 2.6, 0.5 to 1.2, 1.9 to 11.9, 0.3 to 12.9, and 0.3 to 1.4, respectively. The average EF of metals descended in the order As > Ni > Cr > Zn > Cd > Pb > Cu > V, with representative values of 0.6, 1.5, 2.1, 1.4, 0.7, 4.6, 0.8, and 0.7, respectively. Therefore, given that Ni, Cr, and Zn have EFs > 1, the levels of these elements can be further attributed to human activities while the levels of Cd, Cu, Pb, and V, with EFs < 1, can be attributed to the parent rock material or natural weathering processes.

#### 3.3.2. Geo-Accumulation Index (Igeo) 

The geo-accumulation index (Igeo) is often used worldwide to assess the cumulative level of metals and metalloids pollution in soils [39]. In this study, the elemental pollution levels in soils was evaluated using the geo-accumulation index. The results obtained are shown in Figure 2B. The Igeo value ranges for V, Cr, Ni, Zn, Cu, As, Cd, and Pb were −0.9 to 0.5, −0.0 to 1.9, 0.3 to 2.8, −0.4 to 1.9, −1.7 to 0.2, 1.6 to 3.5, −1.6 to 1.4, and −1.6 to 0.9, respectively, with an average value of −0.2, 1.1, 1.5, 0.9, −0.7, 2.5, −0.4, and −0.1, respectively (Figure 2B). 

The Igeo values descended in the following order: As > Ni > Cr > Zn > Pb > V > Cd > Cu. In general, the V, Cd, and Pb mean values are measured as “not polluted,” while the mean value of Zn is indicative of “not polluted to moderately polluted” conditions. In comparison, Cr and Ni’s mean values indicate “moderately polluted” conditions, and the mean value of As exhibits “moderately polluted to heavily polluted” conditions in the investigative areas.

#### 3.3.3. Contamination Factor (CF) and Pollution Load Index (PLI)

**Figure 3:**
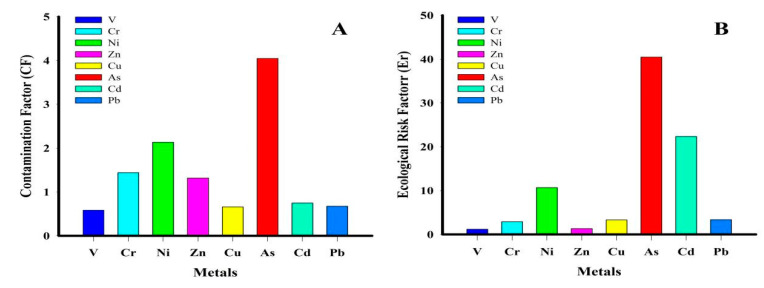

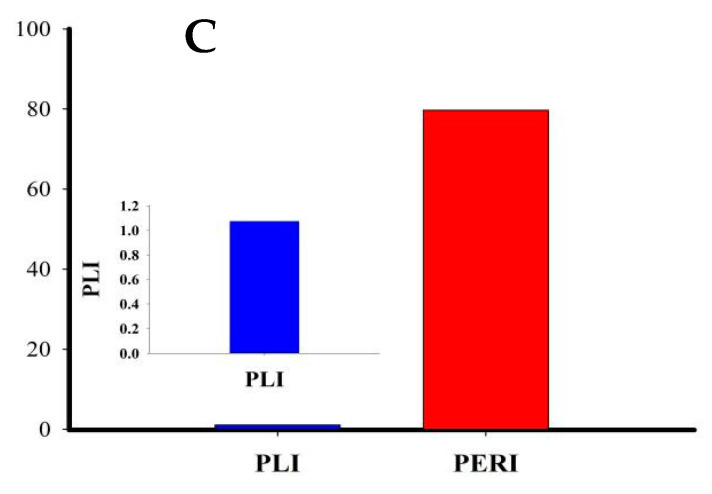
(**A**) Metals contamination factors (CF); (**B**) ecological risk factors (Er); (**C**) pollution load index (PLI) and potential ecological risk (PERI).

The CF and PLI results are shown in Figure 3A,C, respectively. CF indicates elemental pollution level while PLI determines the combined degree of pollution by the different pollutants [17]. The CF values for V, Cr, Ni, Zn, Cu, As, Cd and Pb were 0.9, 1.4, 2.3, 1.3, 0.7, 4.0, 0.6, and 0.7, respectively. The CF values descended in the following order: As > Ni > Cr > Zn > Pb > Cu > V > Cd. Pb, Cu, V, and Cd CFs show a low contamination level. In contrast, Ni, Cr, and Zn CFs indicate a moderate contamination level. Given its relatively higher CF value, the As contamination level is greater than that of the other elements. The PLI values ranged from 0.6 to 1.5, with a mean value of 1.1 (Figure 3C). The results also show that up to 58% (PLI > 1) of the study area may be contaminated with the elements, while 42% (PLI ˂ 1) may not. Together, the CF and PLI results confirm As, Cr, Ni, and Zn contamination in all the studied areas, while more than half of the areas could be contaminated with all the analyzed elements suggesting that these areas should be closely monitored. 

#### 3.3.4. Ecological Risk Factor (Er) and Potential Ecological Risk Index (PERI)

Throughout history, ecological risk indexes have been utilized to illustrate that many biological communities are susceptible to potentially toxic metals [40]. In the present study, the ecological risk factor (Er) and potential ecological risk index (PERI) values were calculated and are presented in Figure 3B. The Er values for V, Cr, Ni, Zn, Cu, As, Cd, and Pb were 1.2, 2.9, 10.7, 1.3, 3.3, 40.5, 16.5, and 3.4, respectively. These results show that all the metals have an Er ≤ 40.46, indicating low to moderate ecological risks [28]. The Er values descended in the following order: As > Cd > Ni > Pb > Cu > Cr > Zn > V. PERI were computed using the ecological risk sum (Er) of individual metals for each soil sample. These values ranged from 45.5 to 132.2, with a mean value of 79.7 (Figure 3C), showing a low potential ecological risk.

### 3.4. Potential Human Health Risks Assessment

In this study, an evaluation of adult and child human health risks of the quantified ingestion, dermal, and inhalation exposure to the metals V, Cr, Zn, Cu, As, Cd, and Pb in the sampled agricultural soils was performed. Noncarcinogenic health risks are shown in Table 2. According to the results, the mean hazard quotient (HQ) values for both adults and children via ingestion (HQingestion), inhalation (HQinhalation), and dermal touch (HQdermal) exposure pathways descend in the following order: HQingestion > HQdermal > HQinhalation indicating that, for both groups, soil ingestion is the major exposure pathway for all the metals analyzed in this study. The HQingestion values for adults for V, Cr, Ni, Zn, Cu, As, Cd, and Pb ranged from 1.13 × 10^−2^ to 2.91 × 10^−2^, 2.22 × 10^−2^ to 8.33 × 10^−2^, 2.03 × 10^−3^ to 1.11 × 10^−2^, 2.02 × 10^−4^ to 9.50 × 10^−4^, 4.89 × 10^−4^ to 1.90 × 10^−3^, 8.04 × 10^−2^ to 2.94 × 10^−1^, 3.04 × 10^−4^ to 1.60 × 10^−2^, and 7.14 × 10^−3^ to 4.13 × 10^−2^ with calculated HQingestion mean values of 1.82 × 10^−2^, 4.82 × 10^−2^, 5.23 × 10^−3^, 5.20 × 10^−4^, 1.08 × 10^−3^, 1.56 × 10^−1^, 1.03 × 10^−3^, and 2.19 × 10^−2^, respectively (Table 2). Similarly, HQingestion values for children for V, Cr, Ni, Zn, Cu, As, Cd, and Pb ranged from 8.05 × 10^−2^ to 2.08 × 10^−1^, 1.59 × 10^−1^ to 5.95 × 10^−1^, 1.45 × 10^−2^ to 7.91 × 10^−2^, 1.44 × 10^−3^ to 6.79 × 10^−3^, 3.49 × 10^−3^ to 1.36 × 10^−2^, 5.74 × 10^−1^ to 2.10, 2.17 × 10^−3^ to 1.14 × 10^−1^, and 5.10 × 10^−1^ to 2.95 × 10^−1^ with calculated HQingestion mean values of 1.30 × 10^−1^, 3.45 × 10^−1^, 3.74 × 10^−2^, 3.71 × 10^−3^, 7.72 × 10^−3^, 1.11, 7.37 × 10^−3^, and 1.57 × 10^−1^, respectively (Table 2). When compared, the mean HQ values for the different exposure routes between adults and children show that all the metal HQ values recorded are higher in the children group than the adult group (Table 2). Our results aligned with previous work in other parts of the world [41,42], indicating that children are at a higher noncancer health risk than adults due to higher soil ingestion potential and relatively lower body mass [43]. 

Furthermore, of all the metals studied, risks due to As ingestion were the highest and posed more significant health hazards to children (HQ = 1.11) than adults (HQ = 1.56 × 10^−1^). According to [44], HQ values of ≤ 1 for a given metal indicate a safe level. However, considering the HQ safe level limits for HQingestion, only As posed a noncarcinogenic health risk and only in children, suggesting that the other metals studied were within acceptable limits in real terms. This finding agrees with [14], who reported that As contamination in a Chinese urban soil sample was potentially harmful to children via oral ingestion, but not adults. Similarly, another study in abandoned gold mines in Ghana reported a significant health risk in children due to As contamination in the area [44]. 

**Figure 4:**
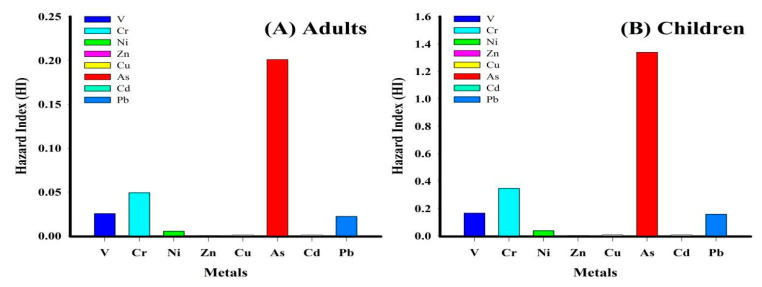
The metals HI: (**A**) adult; (**B**) child.

In Figure 4A,B, the resulting hazard indexes (HI) for V, Cr, Ni, Zn, Cu, As, Cd, and Pb in adults and children are shown. 

The mean HI values of V, Cr, Ni, Zn, Cu, As, Cd, and Pb for adults were 2.54 × 10^−2^, 4.92 × 10^−2^, 5.31 × 10^−3^, 5.30 × 10^−4^, 1.10 × 10^−3^, 2.01 × 10^−1^, 1.04 × 10^−3^, and 2.22 × 10^−2^, respectively, and for children were 1.66 × 10^−1^, 3.47 × 10^−1^, 3.78 × 10^−2^, 3.77 × 10^−3^, 7.80 × 10^−3^, 1.34, 7.39 × 10^−3^, and 1.58 × 10^−1^, respectively. The HI values for adults and children ascended as follows: As > Cr > V > Pb > Ni > Cu > Cd > Zn (Figure 4A,B). An HI value of ˂0.01 indicates the nonhazardous potential of a given element [45]. In the current study, consistent with the HQ results, all HI values, except for As, were ˂0.01, corroborating that the other elements were within safe limits. 

**Figure 5:**
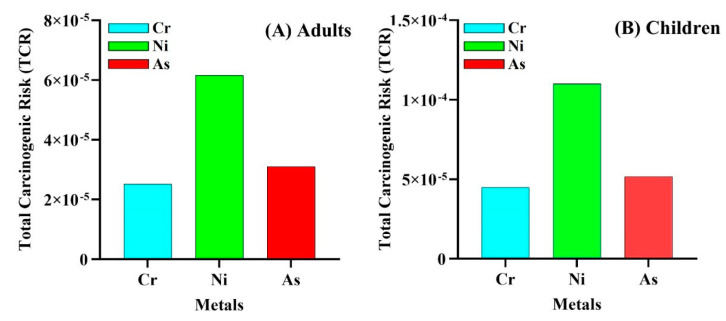
The metals total carcinogenic risks (TCR): (**A**) adult; (**B**) child.

Generally speaking, our findings indicate that children are 6.7 times more likely to experience higher noncarcinogenic effects and are more susceptible to adverse health effects than adults since oral consumption, via hand and mouth, is more common among juveniles [46,47]. Carcinogenic risk (CR) exposures to As, Cr, and Ni were evaluated for adults and children and presented in Table 3 and Figure 5A,B. 

According to USEPA, CR and TCR values below 1 × 10^−6^ are considered negligible and indicate that the subject element is noncarcinogenic, values above 1 × 10^−4^ indicate the carcinogenic potential of metals to humans, and values between 1 × 10^−4^ and 1 × 10^−6^ are considered safe [48]. 

In this study, the CRingestion, CRdermal, and CRinhalation values of the metals decrease as follows: CRingestion > CRdermal > CRinhalation, signifying that ingestion is the main carcinogenic exposure pathway. This result is also consistent with our noncarcinogenic risk results. Further, previous works by Adimalla [49] and Jiang et al. [35] strongly support our results. 

The combined elements (As, Cr, and Ni) total CR values for adults and children were 1.18 × 10^−4^ and 2.06 × 10^−4^, respectively. Both values are larger than the tolerable risk limit (1.00 × 10^−4^). However, further supporting the concept of noncarcinogenic health risk, compared to adults, children demonstrate higher cancer risk when exposed to these elements in the following order: Ni > As > Cr. Additionally, compared to children, As, Cr, and Ni CR values are lower in adults. These results demonstrate that Ni presents a greater carcinogenic health risk to children than adults. Together, our findings suggest that, though both adults and children risk developing cancer over a lifetime due to Ni, As, and Cr contamination, these risks are generally higher in children. Several studies have reported the carcinogenic potential of the elements that were studied in this work. For instance, a recent study by [50] found both adults and children to have significant cancer risk due to exposure to As in rice grown in contaminated agricultural soil. Other studies have also documented varying degrees of As, Ni, and Cr cancer risk potentials to humans in different environmental compartments and edible tissues in other regions of the world [51].

## 4. Conclusions

This study evaluates As, Cd, Cu, Cr, Ni, Pb, V, and Zn contamination levels in Qatar agricultural soils and potential ecological and human health risks according to USEPA’s standard methodologies. The results presented in this work confirm that levels of As, Cr, and Ni in studied soil samples are significantly higher than their corresponding background levels. A human health risks analysis shows that oral ingestion could be the principal exposure pathway in adults and children compared to dermal contact, mouth, and nose inhalation. Although both groups may be at risk when exposed to As, Cr, and Ni contamination, children are more vulnerable and likely to develop cancer. Our findings strongly support authorities’ need to closely monitor potentially toxic elements in agricultural soils and farm produce. Reducing the bioavailability of such elements in soil and developing innovative remediation technologies is needed to limit potential risks to human health. The concentration of these elements in vegetables harvested from the studied areas should be investigated, and more attention be given to the health of children living in surrounding areas. Therefore, future investigations will include a larger number of cultivated soils, larger samples, and primary leafy vegetables grown in the areas analyzed in this study. These will generate more data that is essential for adequately informing new policies and regulating the emerging agricultural sector.

## Figures and Tables

**Table 1:** Sampling locations and their corresponding coordinates.

Sampling Locations	Location Coordinates
1	25°49′28.1″ N 51°19′11.9″ E
2	25°39′09.7″ N 51°22′19.3″ E
3	25°42′37.9″ N 51°21′04.2″ E
4	25°41′13.6″ N 51°25′35.1″ E
5	25°52′17.7″ N 51°22′07.1″ E
6	25°20′00.6″ N 51°08′51.8″ E
7	25°33′47.6″ N 51°09′32.3″ E
8	25°21′17.1″ N 51°13′40.0″ E
9	25°25′00.3″ N 51°22′29.7″ E
10	25°27′22.1″ N 51°22′42.9″ E

**Table 2:** The total metals daily intakes (mg/kg/day) via different exposure pathways and hazard index (HI) (noncarcinogenic health risk) in adults and children.

			V	Cr	Ni	Zn	Cu	As	Cd	Pb
**HQ_ingestion_**	**Adults**	**Mean**	1.82 × 10^−2^	4.82 × 10^−2^	5.23 × 10^−3^	5.20 × 10^−4^	1.08 × 10^−3^	1.56 × 10^−1^	1.03 × 10^−3^	2.19 × 10^−2^
		**Min**	1.13 × 10^−2^	2.22 × 10^−2^	2.03 × 10^−3^	2.02 × 10^−4^	4.89 × 10^−4^	8.04 × 10^−2^	3.04 × 10^−4^	7.14 × 10^−3^
		**Max**	2.91 × 10^−2^	8.33 × 10^−2^	1.11 × 10^−2^	9.50 × 10^−4^	1.90 × 10^−3^	2.94 × 10^−1^	1.60 × 10^−2^	4.13 × 10^−2^
	**Children**	**Mean**	1.30 × 10^−1^	3.45 × 10^−1^	3.74 × 10^−2^	3.71 × 10^−3^	7.72 × 10^−3^	1.11	7.37 × 10^−3^	1.57 × 10^−1^
		**Min**	8.05 × 10^−2^	1.59 × 10^−1^	1.45 × 10^−2^	1.44 × 10^−3^	3.49 × 10^−3^	5.74 × 10^−1^	2.17 × 10^−3^	5.10 × 10^−2^
		**Max**	2.08 × 10^−1^	5.95 × 10^−1^	7.91 × 10^−2^	6.79 × 10^−3^	1.36 × 10^−2^	2.10	1.14 × 10^−1^	2.95 × 10^−1^
**HQ_dermal_**	**Adults**	**Mean**	7.25 × 10^−3^	1.92 × 10^−4^	7.73 × 10^−5^	1.04 × 10^−5^	1.44 × 10^−5^	4.54 × 10^−2^	4.12 × 10^−6^	2.34 × 10^−4^
		**Min**	4.49 × 10^−3^	8.87 × 10^−5^	3.00 × 10^−5^	4.02 × 10^−6^	6.50 × 10^−6^	2.35 × 10^−2^	1.21 × 10^−6^	7.61 × 10^−5^
		**Max**	1.16 × 10^−2^	3.33 × 10^−4^	1.64 × 10^−4^	1.89 × 10^−5^	2.52 × 10^−5^	8.60 × 10^−2^	6.37 × 10^−5^	4.41 × 10^−4^
	**Children**	**Mean**	3.64 × 10^−2^	9.65 × 10^−4^	3.88 × 10^−4^	5.20 × 10^−5^	7.21 × 10^−5^	2.28 × 10^−1^	2.06 × 10^−5^	1.17 × 10^−3^
		**Min**	2.25 × 10^−2^	4.45 × 10^−4^	1.50 × 10^−4^	2.02 × 10^−5^	3.26 × 10^−5^	1.18 × 10^−1^	6.08 × 10^−6^	3.82 × 10^−4^
		**Max**	5.82 × 10^−2^	1.67 × 10^−3^	8.21 × 10^−4^	9.50 × 10^−5^	1.26 × 10^−4^	4.31 × 10^−1^	3.20 × 10^−4^	2.21 × 10^−3^
**HQ_inhalation_**	**Adults**	**Mean**	2.67 × 10^−6^	7.44 × 10^−4^	7.47 × 10^−7^	7.65 × 10^−8^	1.59 × 10^−7^	5.58 × 10^−5^	1.33 × 10^−6^	1.28 × 10^−6^
		**Min**	1.66 × 10^−6^	3.43 × 10^−4^	2.90 × 10^−7^	2.97 × 10^−8^	7.19 × 10^−8^	2.88 × 10^−5^	3.92 × 10^−7^	4.18 × 10^−7^
		**Max**	4.28 × 10^−6^	1.29 × 10^−3^	1.58 × 10^−6^	1.40 × 10^−7^	2.79 × 10^−7^	1.06 × 10^−4^	2.06 × 10^−5^	2.42 × 10^−6^
	**Children**	**Mean**	3.63 × 10^−6^	1.01 × 10^−3^	1.01 × 10^−6^	1.04 × 10^−7^	2.16 × 10^−7^	7.57 × 10^−5^	1.81 × 10^−6^	1.74 × 10^−6^
		**Min**	2.25 × 10^−6^	4.65 × 10^−4^	3.94 × 10^−7^	4.03 × 10^−8^	9.76 × 10^−8^	3.91 × 10^−5^	5.32 × 10^−7^	5.67 × 10^−7^
		**Max**	5.81 × 10^−6^	1.75 × 10^−3^	2.15 × 10^−6^	1.90 × 10^−7^	3.79 × 10^−7^	1.43 × 10^−4^	2.80 × 10^−5^	3.28 × 10^−6^

**Table 3:** The metals carcinogenic risk for adults and children posed by As, Cr, and Ni according to the three exposure pathways.

		Adults			Children		
		Cr	Ni	As	Cr	Ni	As
**CR_ingestion_**	**Mean**	2.48 × 10^−5^	6.10 × 10^−5^	2.40 × 10^−5^	4.43 × 10^−5^	1.09 × 10^−4^	4.29 × 10^−5^
	**Min**	1.14 × 10^−5^	2.37 × 10^−5^	1.24 × 10^−5^	2.04 × 10^−5^	4.23 × 10^−5^	2.22 × 10^−5^
	**Max**	4.29 × 10^−5^	1.29 × 10^−4^	4.54 × 10^−5^	7.66 × 10^−5^	2.31 × 10^−4^	8.12 × 10^−5^
**CR_dermal_**	**Mean**	3.96 × 10^−7^	6.08 × 10^−7^	7.01 × 10^−6^	4.96 × 10^−7^	7.62 × 10^−7^	8.78 × 10^−6^
	**Min**	1.82 × 10^−7^	2.36 × 10^−7^	3.62 × 10^−6^	2.29 × 10^−7^	2.96 × 10^−7^	4.54 × 10^−6^
	**Max**	6.84 × 10^−7^	1.29 × 10^−6^	1.33 × 10^−5^	8.57 × 10^−7^	1.61 × 10^−6^	1.66 × 10^−5^
**CR_inhalation_**	**Mean**	3.06 × 10^−8^	4.75 × 10^−9^	3.55 × 10^−9^	1.04 × 10^−8^	1.61 × 10^−9^	1.21 × 10^−9^
	**Min**	1.41 × 10^−8^	1.84 × 10^−9^	1.84 × 10^−9^	4.79 × 10^−9^	6.26 × 10^−10^	6.23 × 10^−10^
	**Max**	5.29 × 10^−8^	1.01 × 10^−8^	6.73 × 10^−9^	1.80 × 10^−8^	3.41 × 10^−9^	2.28 × 10^−9^

## Data Availability

Data is contained within the article and supplementary materials.

## Supplementary Materials

The following are available online at https://www.mdpi.com/2305-6304/9/2/35/s1, Table S1: Definitions and reference values for parameters used to estimate average daily intake (ADI) for noncarcinogenic and carcinogenic risk, Table S2: Metals reference doses (RfD), Table S3: As, Cr, and Ni cancer slope factors (SF), Table S4: Soil physicochemical parameters, Table S5: Soil ionic contents, Table S6: Descriptive statistics of the soil metals concentrations, and Table S7: The metals correlation coefficients (Pearson’s).
