# Supplementary material for: Ecological and Health Risks Assessment of Potentially Toxic Metals and Metalloids Contaminants: A Case Study of Agricultural Soils in Qatar
Source: Toxics. 2021 Feb 12;9(2):35. doi: 10.3390/toxics9020035 (PMC7918611; doi:10.3390/toxics9020035)
Supplement: Supplementary file 1 [file toxics-09-00035-s001.pdf]

Ecological and Health Risks Assessment of Potentially Toxic Metals and Metalloids Contaminants: A Case Study of Agricultural Soils in Qatar

Mohammed Alsafran [1,2], Kamal Usman [3,*], Hareb Al Jabri [2] and Muhammad Rizwan [4]

Scheme 1. Definitions and reference values for parameters used to estimate average daily intake (ADI) for non-carcinogenic and carcinogenic risk.

Variables	Physical significance and units	Values	
		Adults	Children
IngR	Ingestion rate of soil (mg/day)	100	200
InhR	Inhalation rate of soil (m^3/day)	20	7.6
EF	Exposure frequency (days/year)	350	350
ED	Exposure duration (years)	24	6
BW_A	Average body weight (kg)	56.8	15.9
ET_A	Average exposure time (non-carcinogenic, days)	*ED*×365	*ED*×365
ET_{ca}	Average exposure time (carcinogenic risk, days)	70×365	70×365
ESA_S	Exposed skin surface area (cm^2)	5700	2800
AF_S	Soil to skin adherence factor (mg/cm^2)	0.07	0.2
ABS	dermal absorption factor	As: 0.03<br>Other HMs: 0.001	
EF_p	Particle emission factor (m^3/kg)	1.36×10^9	1.36×10^9

Source: USEPA (1997; 2002; 2009; 2013)

Supplementary Table S2. Metals reference doses (RfD)

	Reference Dose Factor (RfD)		
**Heavy metals**	**Ingestion**	**Dermal**	**Inhalation**
V	7.00E-03	7.00E-05	7.00E-03
Cr	3.00E-03	3.00E-03	2.86E-05
Ni	2.00E-02	5.40E-03	2.06E-02
Zn	3.00E-01	6.00E-02	0.3
Cu	4.00E-02	1.20E-02	4.00E-02
As	3.00E-04	1.23E-04	1.23E-04
Cd	5.00E-04	5.00E-04	5.70E-05
Pb	1.40E-03	5.24E-04	3.52E-03

Supplementary Table S3. As, Cr and Ni cancer slope factors (SF)

	Slope Factor (SF)		
**Heavy metals**	**Ingestion**	**Dermal**	**Inhalation**
**Cr**	5.00E-01	2.00E+00	4.20E+00
**Ni**	1.70E+00	4.25E+00	9.00E-01
**As**	1.50E+00	3.66E+00	1.51E+00

Supplementary Table S4: Soils physicochemical properties (n = 5) ± SEM

Sampling locations	pH	EC (dS m^{-1})	Total Carbon (%)	Total Nitrogen (%)
1	7.5 ± 0.1	120 ± 0.1	5.5 ± 0.3	0.2 ± 0.0
2	7.6 ± 0.1	116 ± 0.4	4.6 ± 0.1	0.2 ± 0.0
3	7.7 ± 0.5	57 ± 0.0	6.2 ± 0.8	0.3 ± 0.0
4	7.5 ± 0.0	87 ± 0.1	8.9 ± 1.2	0.3 ± 0.0
5	7.16 ± 0.1	1,158 ± 2.6	5.6 ± 0.9	0.3 ± 0.0
6	7.3 ± 0.1	175 ± 0.6	4.7 ± 0.1	0.2 ± 0.0

7	7.4 ± 0.0	123 ± 0.4	5.5 ± 0.1	0.1 ± 0.0
8	7.2 ± 0.0	221 ± 0.1	5.4 ± 0.4	0.2 ± 0.0
9	7.1 ± 0	249 ± 0.1	6.5 ± 0.2	0.2 ± 0.0
10	7.1 ± 0.0	273 ± 0.5	5.8 ± 0.2	0.2 ± 0.0

Supplementary Table S5: Soils ionic contents (mg/kg)

	Anions				*Cations*			
Sampling locations	Chloride	Nitrate	Phosphate	Sulfate	Sodium	Potassium	Calcium	Magnesium
1	551 ± 140	135 ± 59	11 ± 7	1980 ± 595	461 ± 82	178 ± 21	684 ± 199	94 ± 16
2	646 ± 274	439 ± 253	44 ± 2	1264 ± 537	522 ± 181	345 ± 138	219 ± 100	144 ± 69
3	174 ± 25	96 ± 31	39 ± 9	554 ± 179	225 ± 27	133 ± 25	234 ± 23	60 ± 5
4	392 ± 90	10 ± 7	19 ± 2	1059 ± 283	447 ± 82	170 ± 18	368 ± 56	87 ± 17
5	16327 ± 4492	1862 ± 587	4 ± 3	8221 ± 1866	10407 ± 2736	758 ± 190	146 ± 737	53 ± 231
6	100 ± 17	8 ± 1	16 ± 6	5714 ± 2562	108 ± 18	96 ± 9	2541 ± 1130	116 ± 26
7	217 ± 37	26 ± 6	11 ± 5	3085 ± 1945	210 ± 28	98 ± 4	1308 ± 812	40 ± 26
8	420 ± 105	190 ± 98	7 ± 2	5924 ± 469	462 ± 96	172 ± 24	2277 ± 260	195 ± 17
9	674 ± 124	26 ± 15	0.5 ± 0.5	6690 ± 524	696 ± 105	145 ± 12	2570 ± 206	205 ± 24
10	401 ± 89	101 ± 76	N.D	8528 ± 2054	428 ± 82	157 ± 16	3616 ± 992	250 ± 66

Supplementary Table S6. Descriptive statistics of the soil metals concentrations (mg/kg)

**Metals**	**V**	**Cr**	**Ni**	**Zn**	**Cu**	**As**	**Cd**	**Pb**
**Minimum**	46.7	39.5	24.1	35.8	11.58	14.2	0.1	5.9
**Maximum**	120.5	148.1	131.2	168.7	44.9	52.3	0.7	34.2
**Mean**	75.3	85.7	61.9	92.3	25.6	27.6	0.2	18.1
**SD**	20.1	24.4	29.1	30.5	7.2	9.7	0.1	7.1
**CV**	26.7	28.4	46.9	33.1	28.1	35.2	57.9	39.1
**Skewness**	0.5	0.2	0.9	0.2	0.2	0.5	2.3	0.4

**Kurtosis**	-0.6	-0.3	-0.3	-0.4	0.0	-0.7	6.6	-0.7
**BGV**	129	59.5	29	70	38.9	6.83	0.41	27

SD: Standard Deviation;
CV: Coefficient of variation
BGV: Background values (Kabata-Pendias and Mukherjee, 2007; Taylor and McLennan, 1995).

Supplementary Table S 7. The metals correlation coefficients (Pearson's)

	**V**	**Cr**	**Ni**	**Zn**	**Cu**	**As**
**V**	1					
**Cr**	0.69***	1				
**Ni**	0.90***	0.75***	1			
**Zn**	0.27	0.20	0.36*	1		
**Cu**	0.66***	0.51***	0.73***	0.73***	1	
**As**	0.14	0.05	0.09	-0.20	-0.08	1
**Cd**	0.20	0.05	0.31*	0.61***	0.48***	0.01
**Pb**	0.22	0.09	0.28	0.73**	0.57***	-0.40***

***Correlation is significant at P ≤ 0.001
**Correlation is significant at P≤ 0.01
*Correlation is significant at P ≤ 0.05
